# Effects of THAP11 on Erythroid Differentiation and Megakaryocytic Differentiation of K562 Cells

**DOI:** 10.1371/journal.pone.0091557

**Published:** 2014-03-17

**Authors:** Xiang-Zhen Kong, Rong-Hua Yin, Hong-Mei Ning, Wei-Wei Zheng, Xiao-Ming Dong, Yang Yang, Fei-Fei Xu, Jian-Jie Li, Yi-Qun Zhan, Miao Yu, Chang-Hui Ge, Jian-Hong Zhang, Hui Chen, Chang-Yan Li, Xiao-Ming Yang

**Affiliations:** 1 Department of Pharmaceutical Engineering, Tianjin University, Tianjin, China; 2 Department of Biochemistry and Molecular Biology, Beijing Institute of Radiation Medicine, Beijing, China; 3 State Key Laboratory of Proteomics, Beijing, China; 4 Department of Hematopoietic Stem Cell Transplantation, Affiliated Hospital to Academy of Military Medical Sciences, Beijing, China; 5 Department of Chemistry, Purdue University, West Lafayette, Indiana, United States of America; 6 Department of Pulmonary Neoplasms Internal Medicine, Affiliated Hospital to Academy of Military Medicine Sciences, Beijing, China; North Carolina State University, United States of America

## Abstract

Hematopoiesis is a complex process regulated by sets of transcription factors in a stage-specific and context-dependent manner. THAP11 is a transcription factor involved in cell growth, ES cell pluripotency, and embryogenesis. Here we showed that THAP11 was down-regulated during erythroid differentiation but up-regulated during megakaryocytic differentiation of cord blood CD34^+^ cells. Overexpression of THAP11 in K562 cells inhibited the erythroid differentiation induced by hemin with decreased numbers of benzidine-positive cells and decreased mRNA levels of α-globin (HBA) and glycophorin A (GPA), and knockdown of THAP11 enhanced the erythroid differentiation. Conversely, THAP11 overexpression accelerated the megakaryocytic differentiation induced by phorbol myristate acetate (PMA) with increased percentage of CD41^+^ cells, increased numbers of 4N cells, and elevated CD61 mRNA levels, and THAP11 knockdown attenuated the megakaryocytic differentiation. The expression levels of transcription factors such as c-Myc, c-Myb, GATA-2, and Fli1 were changed by THAP11 overexpression. In this way, our results suggested that THAP11 reversibly regulated erythroid and megakaryocytic differentiation.

## Introduction

Hematopoietic stem cells (HSCs) differentiate to a number of divergent yet narrowly defined lineages, each giving rise to a specific type of blood cell. The hematopoietic stem cell fate is governed by a complex network of transcription factors. The expression levels and activities of several key transcription factors selectively increase or repress gene expression to determine hematopoietic cell fate [1].

THAP proteins (>100 distinct members in the animal kingdom), a novel family of cellular factors, are defined by the presence of an evolutionarily conserved C2-CH (C-X_2-4_-C-X_35-50_-C-X_2_-H) zinc finger motif of approximately 90 residues with sequence-specific DNA-binding activity [2]. This motif is called the THAP domain [3]. Previous studies have proposed that THAP-containing proteins may play important roles in proliferation, apoptosis, cell cycle, chromosome segregation, chromatin modification, and transcriptional regulation [3], [4]. THAP11, the most recently described member of this human family, is ubiquitously expressed in normal tissues and frequently down-regulated in several human tumor tissues. Enforced expression of THAP11 markedly inhibits cell growth through binding to the promoter of c-Myc and repressing the transcription of c-Myc [5]. Down-regulation of THAP11 by BCR-ABL promotes CML cell proliferation through c-Myc expression [6]. However, immunohistochemical analysis of human colon cancers revealed increased THAP11 expression in both primary tumors and metastases. Knockdown of THAP11 in colon cancer cells resulted in a significant decrease in cell proliferation and THAP11 was found to associate physically with the transcriptional coregulator HCF-1 (host cell factor 1) and recruit HCF-1 to target promoters, then mediating transcriptional regulation [7]. These data suggest that THAP11 is a an important transcriptional and cell growth regulator. The mouse homolog of THAP11 is called Ronin. It has been found to play an essential role in embryogenesis and ES cell pluripotency [8]. Ronin deficiency produces periimplantational lethality and defects in the inner cell mass. Conditional knockout of Ronin prevents the growth of ES cells but enforced expression of Ronin allows ES cells to proliferate without differentiation [8]. Ronin binds to HCF-1, a highly conserved enhancer element located at or immediately upstream of transcription start sites of a subset genes involved in transcription initiation, mRNA splicing, and cell metabolism [9]. These studies suggest that THAP11 is a key transcriptional regulator involved in cell growth and differentiation.

Based on the gene expression file data from several databases, we found that THAP11 is also highly expressed in HSC (short-term HSCs and long-term HSCs), multipotent progenitors (MPP) (http://hscl.cimr.cam.ac.uk/bloodexpress/index.html), and human cord blood CD34^+^CD38^−^ cells (http://xavierlab2.mgh.harvard.edu/EnrichmentProfiler/primary/Expression/212910_at.html). In a study of ontogeny of erythroid gene expression [10], THAP11 is highly expressed in proerythroblasts and down-regulated in basophilic and polyorthochromatic erythroblast. Furthermore, THAP11 is a suppressor of c-Myc which has been reported to play key roles in hematopoietic cell proliferation and differentiation [11]. It is therefore easy to determine whether THAP11 regulates hematopoietic cell differentiation. In this study, we found that THAP11 was up-regulated during erythroid differentiation and down-regulated during megakaryocytic differentiation of cord blood CD34^+^ cells. THAP11 overexpression inhibited the erythroid differentiation of K562 cells induced by hemin, and THAP11 knockdown enhanced erythroid differentiation. Conversely, THAP11 overexpression accelerated the megakaryocytic differentiation induced by phorbol myristate acetate (PMA), and THAP11 knockdown attenuated the megakaryocytic differentiation. These data indicated a reversible role of THAP11 in erythroid differentiation and megakaryocytic differentiation.

## Materials and Methods

### Isolation and culture conditions of human CD34^+^ cells and induction of differentiation

The umbilical cord blood (UCB) samples were collected with the consent of volunteers from the Beijing Haidian Maternal and Child Hospital. Informed consents were confirmed by Institutional Review Board (IRB) in Beijing Haidian Maternal and Child Hospital. Human umbilical cord blood (UCB) was collected, using a clinically approved method, into heparin containing bags immediately after delivery, upon written approval by the mothers. All investigations were approved by IRB of Beijing Haidian Maternal and Child Hospital and Human Research Committees of Beijing Institute of Radiation Medicine. Human CD34^+^ cells were isolated by positive selection from mononuclear cells with anti-CD34 microbeads according to the manufacturer's instructions (StemCell Technologies, Vancouver, BC, Canada). To induce erythroid differentiation, cells were cultured in Iscove-modified Dulbecco medium (IMDM; Invitrogen) supplemented with 20% of serum substitute BIT9500 (StemCell Technologies), 10 U/mL erythropoietin (EPO; Amgen, Thousand Oaks, CA, U.S.), and 10 ng/mL stem cell factor (SCF; R&D Systems Inc., Minneapolis, MN, U.S.). To induce megakaryocytic differentiation, the CD34^+^ cells were cultured in IMDM medium with 10% BIT 9500 Serum Substitute, 50 ng/mL SCF, 50 ng/mL recombinant human thrombopoietin (rh TPO), 50 ng/mL Flt, 10 ng/mL rh interleukin-6 (IL-6), 10 ng/mL IL-3 and 10 ng/mL IL-11 (R&D Systems Inc).

### Cell culture and induced differentiation

Human chronic myelogenous leukemia cell line K562 cells and TF-1 cells were purchased from American Type Culture Collection (Manassas, VA). These cells were cultured in RPMI 1640 medium (Gibco Invitrogen, CA, U.S.) with 10% fetal calf serum at 37°C and 5% CO_2_. When differentiation was induced, exponentially grown K562 cells were treated with 40 µM hemin (Sigma–Aldrich, St. Louis, MO, U.S.) as described previously [12]. To induce megakaryocytic differentiation, K562 cells were treated with 10 nM PMA (Sigma–Aldrich) for the desired lengths of time.

Erythroid differentiation of K562 cells was assessed by the benzidine cytochemical test and the benzidine-positive cells represented more mature erythroid cells [12]. mRNA levels of the erythroid genes α-globin (HBA) and glycophorin A (GPA) were detected using real-time PCR. Megakaryocytic differentiation of K562 cells was assessed by analyzing CD41 positive cells and assessing the relative number of 4N cells [13]. The CD61 mRNA level was detected using real-time PCR.

### Lentivirus vector construction and production

For the lentivirus-mediated THAP11 overexpression, full-length THAP11 with Myc-tag was cloned into the pBPLV vector to generate pBPLV-THAP11(Myc) recombinant vector expressing Myc-tagged THAP11 protein. For lentivirus-mediated RNA interference, two siRNA oligos against THAP11 were synthesized using GenePharma Biotechnology, The sequences are provided in Table S1. The siRNAs were cloned into a psicoR-GFP vector to generate siTHAP11 lentivirus. A negative control siRNA was cloned into psicoR-GFP as a control. For production of lentivirus, HEK293 cells were cotransfected with transfer vectors pBPLV-THAP11 or psicoR-GFP-siTHAP11 with packaging vectors pLP1, pLP2, and pLP-VSVG. Lentiviruses were harvested 72 hours after transfection. GFP-positive cells were sorted using a fluorescence-activated cell sorter (FACS; BD Biosciences).

### Western blot analysis

Cells were lysed with M-PER Mammalian Protein Extraction Reagent (Pierce, Rockford, IL, U.S.). Then, Western blot analysis was performed according to standard procedures. Antibodies were used at the following concentrations: THAP11 (R&D), 1∶1000; GAPDH (Santa Cruz), 1∶1000. Chemiluminescent detection was conducted using supersignal substrate according to the manufacturer's specifications (Pierce, Rockford, IL, U.S.).

### Reverse transcription-PCR (RT-PCR) and quantitative real-time RT-PCR

Total RNA was reverse-transcribed and amplified using reverse transcription and PCR kits, respectively (Promega Corp., Madison, WI, U.S.). Real-time RT-PCR was performed by Bio-Rad IQ5 (Bio-Rad, U.S.). The abundance of mRNA of each gene was normalized to GAPDH. The sequences of the primers are provided in Table S1.

### Chromatin immunoprecipitation (ChIP)

Cultured cells were cross-linked with 1% formaldehyde for 10 min at room temperature and quenched in 125 mM glycine. The cells were washed in phosphate buffered saline (PBS) then lysed in buffer containing 1-0.1% of SDS. Sonicated DNA fragments were incubated overnight at 4°C with 5 µg of ChIP grade antibodies. Bound material was precipitated with ProteinA agarose beads (upstate) at 4°C for 5 hours followed by washing and de-crosslinking for subsequent analysis. Samples were treated with 10 µg RNaseA for 20 min at 37°C, and DNA was recovered using the QIAquick PCR Purification kit (QIAGEN). Enrichment of the ChIP sample over Input was confirmed by PCR. Antibodies used were as follow: Anti-THAP11 (R&D),, normal sheep IgG (Santa Cruz).

### Statistical analysis

Error bars representing the Means ± standard deviations were determined from three separate experiments. All statistical analysis was performed by one-way analysis of variance (ANOVA) followed by the two-sided Dunnett post-hoc tests.

## Results

### Regulation of THAP11 during erythroid differentiation and during megakaryocytic differentiation of cord blood CD34^+^ cells

**Figure 1:**
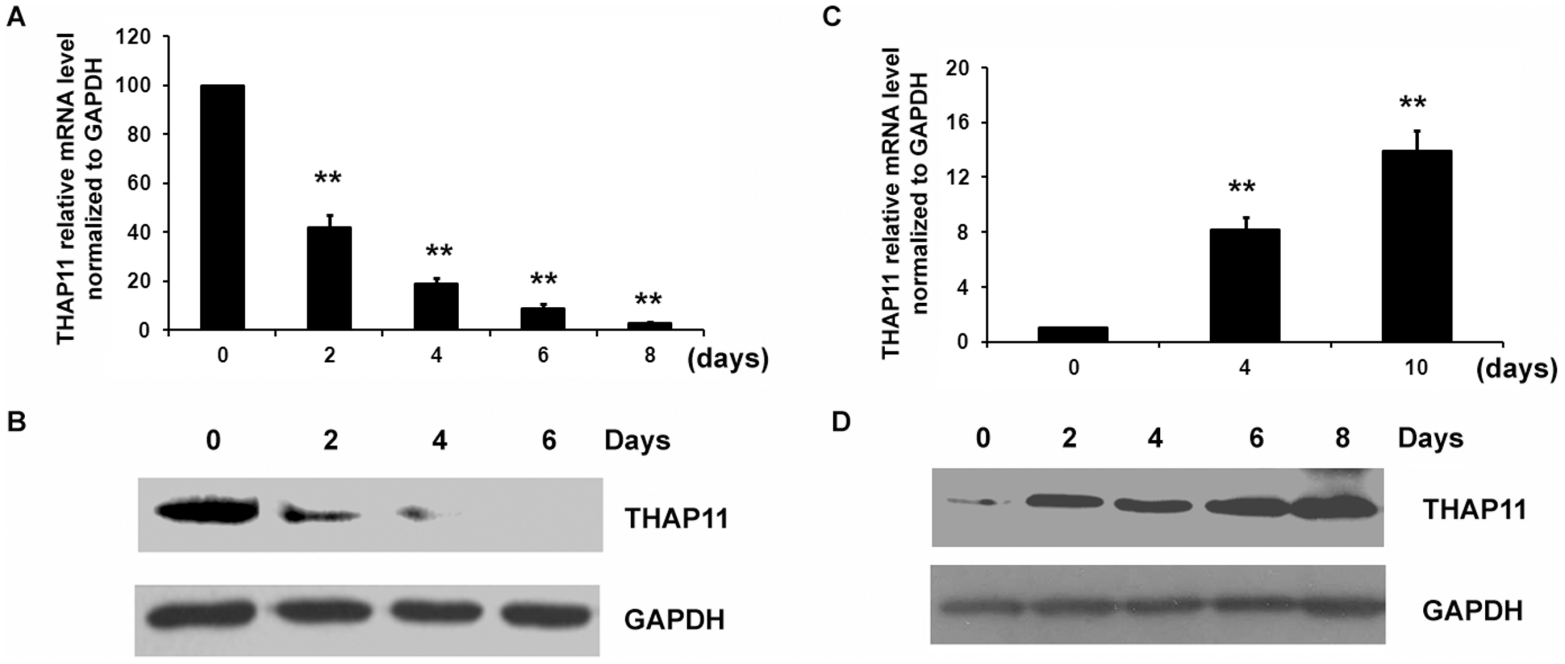
THAP11 expression profile during differentiation of cord blood CD34^+^ cells. (A) CD34^+^ cells were cultured in the presence of EPO for the indicated lengths of time and the expression level of THAP11 was detected using real-time PCR and Western blot analysis. (B) For the Western blot analysis, 80 µg protein was loaded. (C) CD34^+^ cells were cultured in the presence of TPO for the indicated lengths of time and the expression level of THAP11 was detected using real-time PCR and Western blot analysis. (D) For the Western blot analysis, 10 µg protein were loaded. Real-time PCR results were expressed as fold induction compared to cells at day 0 and normalized to GAPDH mRNA. Each bar represents the mean ± SD for three independent experiments. The statistical difference between the samples is given using * *P*≤0.05 or ** *P*≤0.001.

We investigated the expression profile of THAP11 during erythroid differentiation and megakaryocytic differentiation of CD34^+^ cells. Glycophorin A (GPA) was used as a marker of mature erythrocytes and CD41 was used as a marker of mature megarkaryocytes. The THAP11 expression level was measured using real-time PCR and Western blot analysis. In the culture system containing EPO, CD34^+^ cells underwent erythroid differentiation with increased numbers of GPA^+^ cells (Fig. S1–A). On day 6, the number of GPA^+^ cells had increased by 62.4% from the initial level, suggesting that the erythroid differentiation had been induced. As shown in Fig. 1A–B, THAP11 was down-regulated significantly during erythroid differentiation of CD34^+^ cells and was barely detectable on day 6 after EPO induction. In the culture system containing TPO, the number of CD41^+^ cells began to increase on day 2. On day 6, the number of CD41^+^ cells was 37.3% higher than the initial value. On day 8 after TPO induction, there were 58.2% more CD41^+^ cells than there had been on the day of treatment, suggesting that CD34^+^ cells underwent megakaryocytic differentiation (Fig. S1–B). As shown in Fig. 1C–D, during the megakaryocytic differentiation of CD34^+^ cells, the THAP11 expression level was up-regulated. Differences in THAP11 expression in hematopoietic cells during differentiation indicated a potential role of THAP11 in erythroid and megakaryocytic differentiation.

### Effects of THAP11 on erythroid differentiation of K562 cells induced by hemin

**Figure 2:**
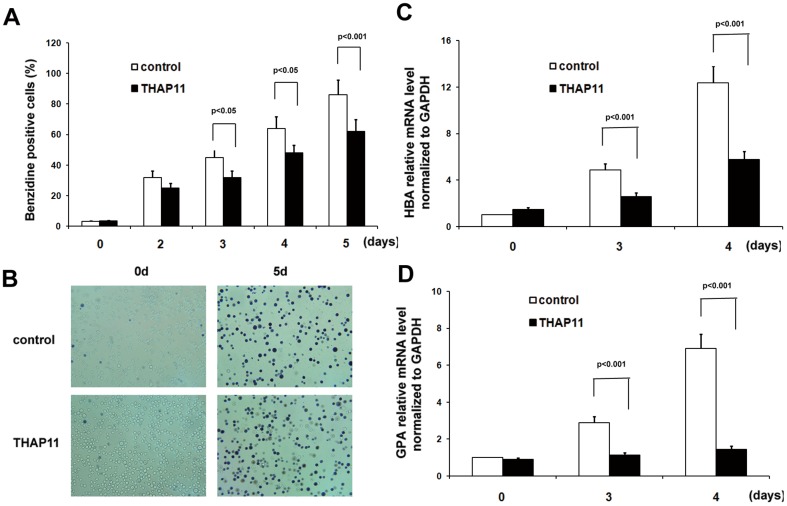
THAP11 overexpression leads to inhibition of hemin induced erythroid differentiation of K562 cells. (A) THAP11 lentivirus infected K562 cells (THAP11-LV) and control cells were treated with 40 µM hemin for the indicated lengths of time and the benzidine-positive cells were counted. The pictures of cells treated with hemin for 5 days were shown in (B). (C) HBA mRNA level and (D) GPA mRNA level were detected using real-time PCR analysis. Real-time PCR results were expressed as fold induction relative to control cells at day 0 and normalized to GAPDH mRNA.

Human erythroleukemia cell line K562 can be differentiated into erythroid cells by treatment with hemin and into megakaryocytic cells by treatment with PMA [7]. In this way, K562 cells were used as a model cell line for the studies of erythroid or megakaryocytic differentiation. The expression level of THAP11 changed significantly during erythroid and megakaryocytic differentiation of K562 cells and the expression profile of THAP11 during erythroid differentiation was very similar to that in CD34^+^ cells (Fig. S2). To gain insight into the role of THAP11 in human erythroid differentiation, we first overexpressed THAP11 by lentivirus transduction of K562 cells. K562 cells were transduced with a THAP11cDNA-Myc expression cassette upstream of an IRES-GFP element (THAP11-LV) and in parallel with a control empty vector (control). The efficiency of transduction was assayed by monitoring GFP expression. GFP^+^ cells were purified by FACS. The expression of exogenous THAP11 was verified by Western blot analysis (Fig. S3). Overexpression of THAP11 in K562 cells resulted in decreased cell proliferation, but no obvious apoptosis was observed, which was consistent with the previous study [6] (data not shown). Then the cells were treated with 40 µM hemin for the indicated length of time and the number of benzidine-positive cells was determined (Fig. 2A). No significant difference in the relative number of benzidine-positive cells was detected in K562 cells infected with control lentivirus and THAP11 lentivirus before hemin stimulation. After treatment with hemin, control K562 cells showed significant increases in the number of benzidine-positive, and THAP11-LV cells showed significantly fewer benzidine-positive cells (Fig. 2A–B). The expression of endogenous and overexpressed THAP11 was shown as Fig. S4. The erythroid genes α-globin (HBA) and glycophorin A (GPA) mRNA levels were also much lower in THAP11-LV cells compared to the control cells (Fig. 2C–D).

**Figure 3:**
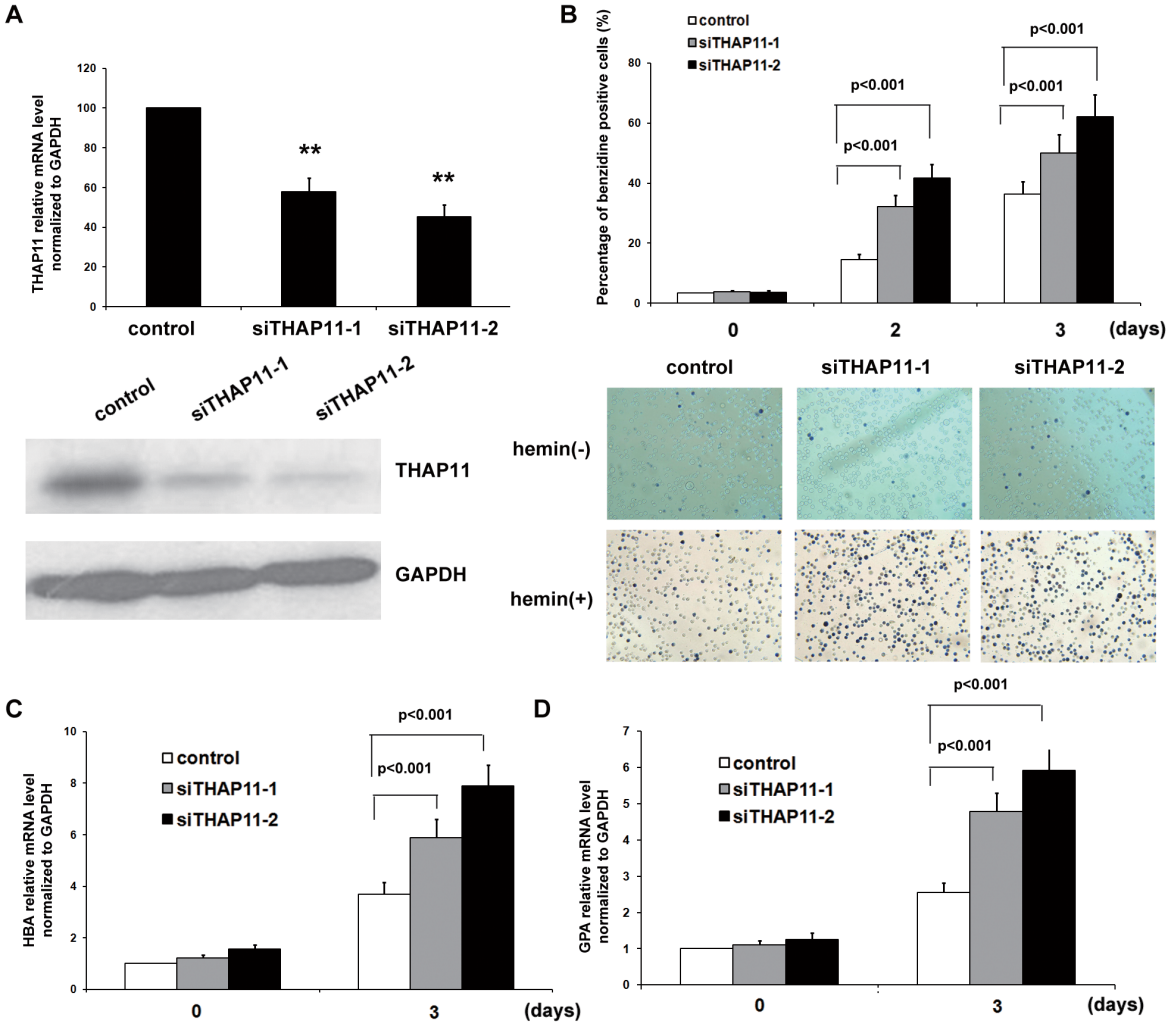
THAP11 knockdown enhances hemin induced erythroid differentiation of K562 cells. (A) K562 cells were infected with control lentivirus or THAP11 RNAi lentivirus (siTHAP11-1 and siTHAP11-2) and then the GFP positive cells were sorted. The THAP11 expression level was detected by real-time PCR (upper panel) and Western blot analysis (lower panel). Then the cells were treated with 40 µM hemin for the indicated lengths of time. (B) Benzidine-positive cells were counted. (C) HBA and (D) GPA mRNA levels were measured using real-time PCR analysis.

To investigate the effects of endogenous THAP11 on erythroid differentiation, K562 cells were infected with lentivirus-based THAP11 siRNA or universal scramble siRNA (control). Then the GFP-positive cells were purified and treated with hemin for the indicated lengths of time. As shown in Fig. 3A, the THAP11 siRNA lentivirus decreased the endogenous THAP11 mRNA and protein levels relative to the control cells. Under hemin treatment, siTHAP11 cells showed higher numbers of benzidine-positive cells than control cells did (Fig. 3B). The HBA (Fig. 3C) and GPA mRNA levels (Fig. 3D) were significantly higher among treated cells than among controls. To confirm the effect of THAP11 on erythroid differentiation, another human erythroleukemia cell line, TF-1 cells, which can be induced to erythroid differentiation by EPO, was used. Similar results were observed (Fig. S5)

These results suggest that enforced expression of THAP11 inhibits hemin-induced erythroid differentiation of K562 cells.

### Effects of THAP11 on rate of megakaryocytic differentiation among K562 cells induced by PMA

**Figure 4:**
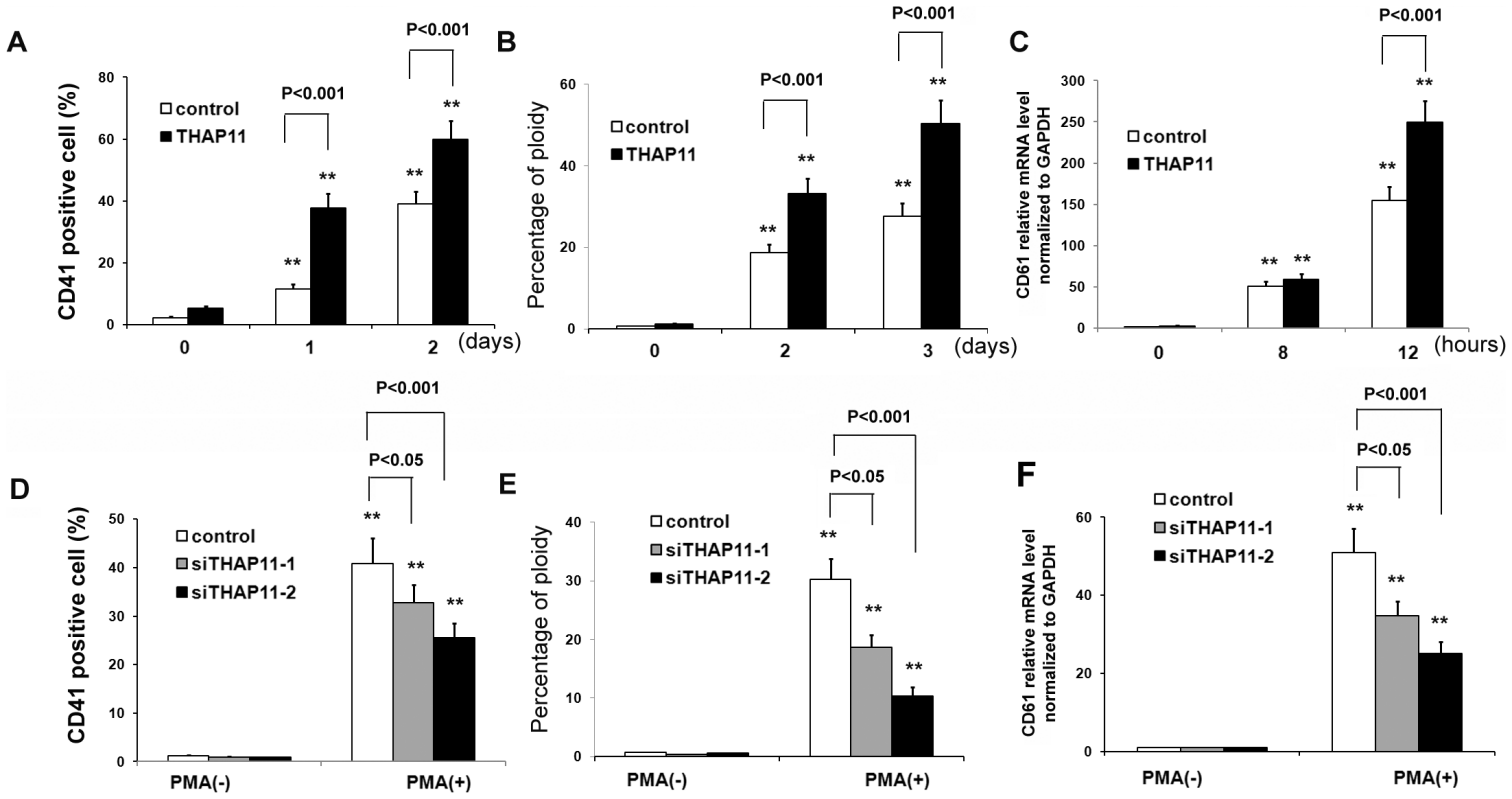
THAP11 accelerates megakaryocytic differentiation of K562 cells induced by PMA. THAP11 lentivirus infected K562 cells (THAP11-LV) and control cells were treated with 10 nM PMA for the indicated lengths of time. CD41^+^ cells (A) and percentage of 4N cells (B) were analyzed using FACS. The CD61 mRNA level was measured by real-time PCR analysis (C). (D) THAP11 siRNA lentivirus infected-K562 cells or control K562 cells were treated with 10 nM PMA for 3 days. Then CD41^+^ cells and the percentage of 4N cells (E) were analyzed using FACS. (F) The CD61 mRNA level was measured using real-time PCR analysis.

In order to determine the effects of enforced expression of THAP11 on megakaryocytic differentiation among K562 cells, the THAP11-LV K562 cells and control cells were cultured in medium containing 10 nM PMA for the indicated lengths of time. Then CD41^+^ cells were detected. As shown in Fig. 4A, in control cells, the relative numbers of CD41^+^ cells increased to 11.4% at 1 day and to about 38.9% at 2 days after PMA treatment. However, in THAP11-LV cells, the relative numbers of CD41^+^ cells increased to 37.6% at 1 day and 59.8% at 2 days after treatment.

Ploidy analysis by flow cytometry showed that the peak corresponding to 4N cells was increased in both control cells and THAP11-LV cells with PMA induction. The peaks corresponding to 4N cells and the relative number of 4N THAP11-LV cells after 1 day and 2 days of treatment were higher than in control cells (Fig. 4B). The CD61 mRNA level was also evaluated and similar results was observed (Fig. 4C).

We next investigated megakaryocytic differentiation in K562 cells infected with THAP11 siRNA lentiviruses and in control cells. Consistently, inhibition of THAP11 expression led to reduced megakaryocytic differentiation of K562 cells induced by PMA with lower numbers of CD41^+^ cells (Fig. 4D) and 4N cells (Fig. 4E) and lower expression level of CD61 mRNA (Fig. 4E).

These results indicate that enforced expression of THAP11 causes increased megakaryocytic differentiation among K562 cells.

### Effects of THAP11 overexpression on mRNA expression levels of several hematopoietic transcription factors

Previous studies suggest that histone deacetylase activity regulates hematopoietic differentiation. We further investigated the effect of THAP11 on histone deacetylation in K562 cells during megakaryocytic differentiation. The data suggested that the acetylation levels of H3K9 and H3K18 were not affected by THAP11 overexpression (Fig. S6).

**Figure 5:**
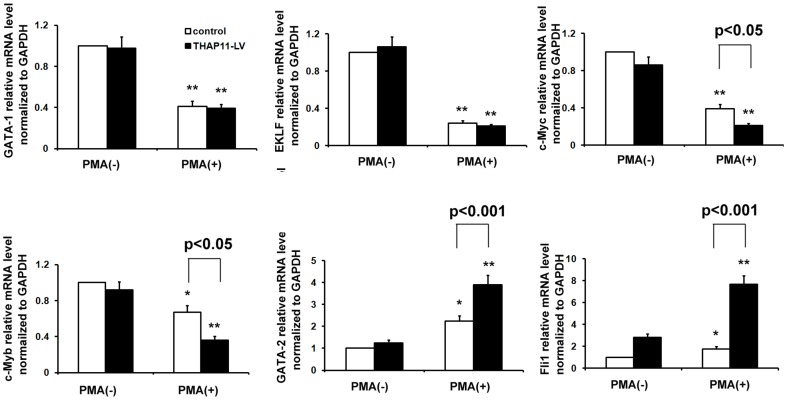
Alteration of expression levels of several hematopoietic transcription factors in THAP11-overexpressing K562 cells. K562 cells infected with THAP11-lentiviruses (THAP11-LV) and control cells were treated with 10 nM PMA for 72 hours. Then total RNA was extracted for real-time PCR analysis. Real-time PCR results were expressed as fold induction relative to cells at day 0 and normalized to GAPDH mRNA. Each bar represented the mean ± SD for three independent experiments. The statistical difference between the samples was demonstrated as * *P*≤0.05 or ** *P*≤0.001.

Several hematopoietic transcription factors, including GATA-1, GATA-2, c-Myb, EKLF, Fli-1, and c-Myc, have been shown to be critical to the commitment of HSC to megakaryocytic and erythroid lineages [14]. For this reason, we analyzed whether THAP11 over-expression could modify the expression levels of these transcription factors. As shown in Fig. 5, PMA treatment induced significant down-regulation of EKLF, GATA-1, c-Myc, and c-Myb but it up-regulated GATA-2 and Fli1. In THAP11-LV cells during PMA-induced megakaryocytic differentiation, the expression levels of c-Myc and c-Myb were much lower than in control cells, but the levels of GATA2 and Fli1 were much higher. THAP11-LV cells showed GATA-1 and EKLF mRNA levels similar to those observed in control cells. We also investigated the mRNA level of these transcription factors during hemin-induced erythroid differentiation. As shown in Fig. S7, with hemin treatment THAP11 overexpression led to down-regulation of c-Myc and up-regulation of Fli1,while the expression levels of GATA-1, GATA-2, c-Myb and EKLF were not affected significantly. Our previous study suggested that THAP11 suppresses c-Myc expression through binding to the promoter of c-Myc, then we investigated whether THAP11 binds to the promoters of GATA-2, Fli1 and c-Myb. Bioinformatic analysis suggested that in all the promoters of these genes, there are several potential THAP11 binding sites (Fig. S8). ChIP analysis was performed using specific anti-THAP11 antibody and normal sheep IgG as negative control. The sequence from −710/−463 region of c-Myc promoter was used as positive control. As shown in Fig. S9, THAP11 was found to occupy at the promoters of GATA-2, Fli1, c-Myb and c-Myc genes, but not at a random site that was derived from the promoter region of the human GAPDH genome. These results indicate that THAP11 might regulate the expression levels of several key hematopoietic transcription factors directly.

## Discussion

In the present study, we show that THAP11 plays dual actions on erythroid differentiation and megakaryocytic differentiation. Using K562 cells as a model, we found that overexpression of THAP11 suppressed hemin induced erythroid differentiation of K562 cells with fewer benzidine-positive cells and lower mRNA levels of HBA and GPA. In contrast, THAP11 overexpression led to enhanced megakaryocytic differentiation induced by PMA with increased numbers of 4N cells, elevated numbers of CD41^+^ cells, and a higher level of CD61 mRNA. The expression levels of several hematopoietic transcription factors, including GATA-2, c-Myc, Fli1, and c-Myb, were changed by THAP11 overexpression. In this way, our results suggest that THAP11 reversibly regulates erythroid and megakaryocytic differentiation.

Hematopoiesis is a complex process regulated by sets of transcription factors in a stage-specific and context-dependent manner. Lineage commitment from HSC involves the activation of specific gene programs and concomitant suppression of multipotential and alternate lineage gene programs [15]. Bipotent committed precursors such as MEPs undergo further fate decisions by being directed toward either the erythroid or megakaryocytic branch, respectively [16]. It has been suggested that these dual-fate specification processes of HSCs and hematopoietic progenitors are largely controlled by fate-specific transcription factors and are regulated by stimulatory or repressive signals produced by BM niche cells [17]. Despite many efforts, the transcription factors responsible for guiding the fate determination of HSCs and hematopoietic progenitors have not yet been fully identified. As our previous study describes, THAP11 is a transcriptional factor that suppresses cell growth through repressing the transcription of c-Myc. c-Myc has been widely reported to play a key role in hematopoietic differentiation. In mammalian erythroid cells, an important dose-dependent function of Myc is revealed in regulating terminal maturation [18] and down-regulation of Myc is essential for terminal erythroid maturation. During TPA-induced megakaryocytic differentiation of K562 cells, c-Myc expression is rapidly down-regulated [19]. c-Myc^−/−^ mice show significantly increased numbers of megakaryocytic progenitors and mature megakaryocytes in bone marrow and spleens, together with blocked differentiation of erythrocytes at the erythroid progenitor stage, suggesting an essential role c-Myc-mediated control of cell fate in megakaryocyte-erythrocyte progenitors [20]. This phenotype is very similar to that of THAP11 overexpressing K562 cells since THAP11 overexpression leads to inhibition of erythroid differentiation and accelerated megakaryocytic differentiation, together with reduced expression level of c-Myc. Furthermore, c-Myc is also differentially expressed during the differentiation of MEPs to megakaryocytes and erythrocytes. Megakaryocytic progenitors (Mk-Ps) express lower levels of c-Myc than erythrocytic progenitors (Ery-Ps). The expression level of c-Myc is significantly down-regulated during megakaryocyte maturation and up-regulated during the differentiation of Ery-Ps to erythrocytic blasts (Ery-Bs) [20]. In contrast, THAP11 is up-regulated during megakaryocytic differentiation and down-regulated during erythroid differentiation in primary CD34^+^ cells. This suggests that THAP11 plays a role opposite to that of c-Myc in hematopoietic differentiation. Whether THAP11 regulates megakaryocytic-erythroid differentiation in primary hematopoietic stem cells and whether THAP11 suppresses c-Myc expression *in vivo* remain to be determined.

The expression of transcription factors c-Myb, GATA-2, and Fli1 is also appeared to be modified by THAP11 overexpression. A previous study reported that c-Myb silencing in human CD34^+^ hematopoietic stem/progenitor cells increased commitment capacity toward the macrophage and megakaryocyte lineages but impaired erythroid differentiation [21] suggesting that c-Myb regulates erythroid differentiation in a positive manner. GATA-2 is a key transcription factor in controlling cell fate outcome within the stem and early progenitor cell compartments and plays an important role in hematopoietic commitment [22] [23]. Overexpression of GATA-2 overexpression in hematopoietic cells inhibits erythroid maturation [24], [25] while inducing megakaryocytic differentiation [24] [26]. Fli1 is a suppressor of erythroid differentiation and induces megakaryocytic differentiation in human hematopoietic cells [27] [28]. Fli-1 gene-targeted mice show defective megakaryopoiesis and abnormal erythroid development, suggesting an important role of Fli1 in megakaryocytic lineage commitment. Our data demonstrate that THAP11 overexpression inhibited the expression of c-Myb while enhancing the expression of GATA-2 and Fli1. So the converse role of THAP11 on erythroid and megakaryocytic differentiation seems to be associated with alterations of transcription factors such as up-regulation of the megakaryocytic related genes and repression of the genes related to erythroid differentiation. More interestingly, we found that THAP11 can bind to the promoter regions of these genes, suggesting that these genes might be direct target genes of THAP11, However, further detailed investigations such as EMSA and promoter activity assay are needed to confirm this issue.

Although we showed that THAP11 was up-regulated during megakaryocytic differentiation of primary human CD34^+^ cells and overexpression of THAP11 in K562 cells increased the megakaryocytic differentiation of K562 cells, it is very interesting that the expression profile of THAP11 during megakaryocytic differentiation in K562 cells is not similar to that in CD34^+^ cells. THAP11 expression first decreased after PMA treatment in K562 cells from 24 hrs to 48 hrs, and then increased at 72 hrs time point. This discrepancy raised the possibility that THAP11 might be not essential for megakaryocyte development *in vivo*. Overexpression of Tescalcin in K562 cells was associated with an increase in the population of cells that expressed GPIIb and other markers of megakaryocyte differentiation [29]. However, Tescalcin knockout mice showed normal numbers of megakaryocytes, normal megakaryocyte morphology, normal polyploidization, normal expression of Fli-1 and normal platelet, suggesting that Tescalcin is not essential for megakaryocyte development *in vivo* [30]. THAP11 shows similar expression profile with Tescalcin during megakaryocytic differentiation in K562 cells, so it remains a matter of concern that whether THAP11 is a key factor for megakaryocytic differentiaiton *in vivo*. To confirm the role of THAP11 in megakaryocytic development, further *in vivo* models are needed.

In this study, we provide the first line of evidence that THAP11 reversibly regulates erythroid and megakaryocytic differentiation in K562 cells. Our data suggest a novel role for the THAP11 protein in hematopoietic differentiation.

## Supporting Information

Figure S1 **Erythroid and megakaryocytic differentiation of CD34^+^ cells.** Human cord blood CD34^+^ cells were cultured in the presence of (A) EPO or (B) TPO for the indicated time and then cells were stained with PE- GlyA or PE-CD41 antibody for flow cytometry analysis.
(DOCX)

Figure S2 **THAP11 expression profile during differentiation of K562 cells.** K562 cells were treated with (A) 40 µM hemin or (B) 10 nM PMA for the indicated time. Then the THAP11 expression level was analyzed using real-time PCR (upper panel) and Western blot analysis (lower panel). Real-time PCR results were expressed as fold induction relative to cells at day 0 and normalized to GAPDH mRNA. Each bar represented the mean ± SD for three independent experiments. The statistical difference between the samples was demonstrated as * *P*≤0.05 or ** *P*≤0.001. For Western blot analysis, GAPDH was used as internal control.
(DOCX)

Figure S3 **THAP11 expression level in lentivirus-infected K562 cells.** K562 cells were infected with control lentivirus (control) or THAP11 lentivirus (THAP11-LV) for twice in 48 hours. Then the GFP^+^ cells were sorted for Western blot analysis. GAPDH was used as internal control. exTHAP11: overexpressed THAP11; enTHAP11: endogenous THAP11.
(DOCX)

Figure S4 **THAP11 expression levels in lentivirus-infected K562 cells during hemin-induced megakaryocytic differentiation.** K562 cells were infected with control lentivirus (control) or THAP11 lentivirus (THAP11-LV) and GFP^+^ cells were purified. Then the cells were treated with 40 µM hemin for the indicated length of time and the THAP11 expression level was analyzed using Western blot analysis with anti-THAP11 antibody. GAPDH was used as internal control. exTHAP11: overexpressed THAP11; enTHAP11: endogenous THAP11.
(DOCX)

Figure S5 **THAP11 inhibits erythroid differentiation of human erythroleukemia cell line TF-1 induced by EPO.** (A) TF-1 cells were infected with THAP11 lentivirus or control lentivirus and then cultured in the presence of 0.5 ng/ml GM-CSF and 5 IU/ml EPO for the indicated time. Then the benzidine positive cells were counted. The (B) HBA and (C) GPA mRNA levels were analyzed using real-time PCR. (D) THAP11 siRNA lentiviruses or control lentivirus were infected into TF-1 cells and cultured in the presence of 0.5 ng/ml GM-CSF and 5 IU/ml EPO for the indicated time. Then the benzidine-positive cells were counted. The (E) HBA and (F) GPA mRNA levels were analyzed using real-time PCR.
(DOCX)

Figure S6 **The effect of THAP11 on H3 acetylation during PMA-indced megakaryocytic differentiation of K562 cells.** K562 cells infected with THAP11-lentiviruses (THAP11-LV) and control cells were treated with 10 nM PMA for the indicated length of time. Then total cell lysates were prepared for analyzing the acetylation level of histone H3 using specific antibodies against acetylated-H3K9 and acetylated-H3K18. GAPDH was used as an internal control.
(DOCX)

Figure S7 **Alteration of expression levels of several hematopoietic transcription factors in THAP11-overexpressing K562 cells with hemin treatment.** K562 cells infected with THAP11-lentiviruses (THAP11-LV) and control cells were treated with 40 µM hemin for 72 hours. Then total RNA was extracted for real-time PCR analysis. Real-time PCR results were expressed as fold induction relative to cells at day 0 and normalized to GAPDH mRNA. Each bar represented the mean ± SD for three independent experiments. The statistical difference between the samples was demonstrated as * *P*≤0.05 or ** *P*≤0.001.
(DOCX)

Figure S8 **Bioinformatic analysis of the promoter sequences of GATA2 (A), c-Myb (B) and Fli1 (C) genes.** The potential THAP11 binding consensus sequences are framed.
(DOCX)

Figure S9 **THAP11 occupies the promoter regions of GATA2, c-Myb and Fli1 genes.** (A) ChIP at the promoter region of indicated genes using anti-THAP11 antibody and IgG as the control in K562 cells. A random probe was used from human GAPDH genome. (B) The results are averages of at least 3 independent experiments. Error bars represent the mean ± SD. Statistical significance was determined by comparing the occupancy of specific antibodies and the IgG control; *P≤0.05, ** p<0.001.
(DOCX)

Table S1 **Sequences of primers used in the present study.**
(DOC)
