# Supplementary material for: Effects of THAP11 on Erythroid Differentiation and Megakaryocytic Differentiation of K562 Cells
Source: PLoS One. 2014 Mar 17;9(3):e91557. doi: 10.1371/journal.pone.0091557 (PMC3956667; doi:10.1371/journal.pone.0091557)
Supplement: Figure S1 — Erythroid and megakaryocytic differentiation of CD34+ cells. Human cord blood CD34+ cells were cultured in the presence of (A) EPO or (B) TPO for the indicated time and then cells were stained with PE- GlyA or PE-CD41 antibody for flow cytometry analysis. (DOCX) [file pone.0091557.s001.docx]

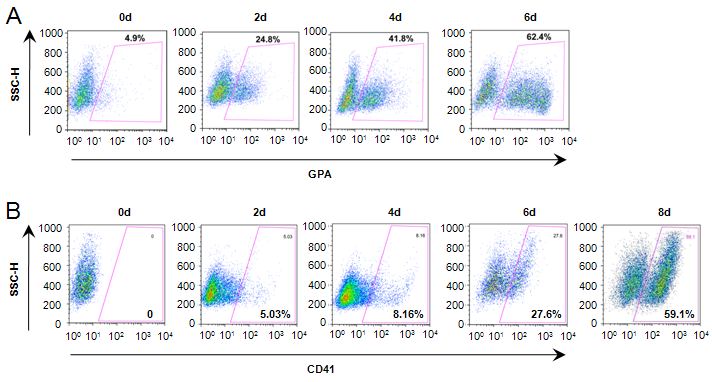


**Fig. S1 Erythroid and megakaryocytic differentiation of CD34^+^ cells.** Human cord blood CD34^+^ cells were cultured in the presence of (A) EPO or (B) TPO for the indicated time and then cells were stained with PE- GlyA or PE-CD41 antibody for flow cytometry analysis.
