# Supplementary material for: Effects of THAP11 on Erythroid Differentiation and Megakaryocytic Differentiation of K562 Cells
Source: PLoS One. 2014 Mar 17;9(3):e91557. doi: 10.1371/journal.pone.0091557 (PMC3956667; doi:10.1371/journal.pone.0091557)
Supplement: Figure S2 — THAP11 expression profile during differentiation of K562 cells. K562 cells were treated with (A) 40 µM hemin or (B) 10 nM PMA for the indicated time. Then the THAP11 expression level was analyzed using real-time PCR (upper panel) and Western blot analysis (lower panel). Real-time PCR results were expressed as fold induction relative to cells at day 0 and normalized to GAPDH mRNA. Each bar represented the mean ± SD for three independent experiments. The statistical difference between the samples was demonstrated as * P≤0.05 or ** P≤0.001. For Western blot analysis, GAPDH was used as internal control. (DOCX) [file pone.0091557.s002.docx]

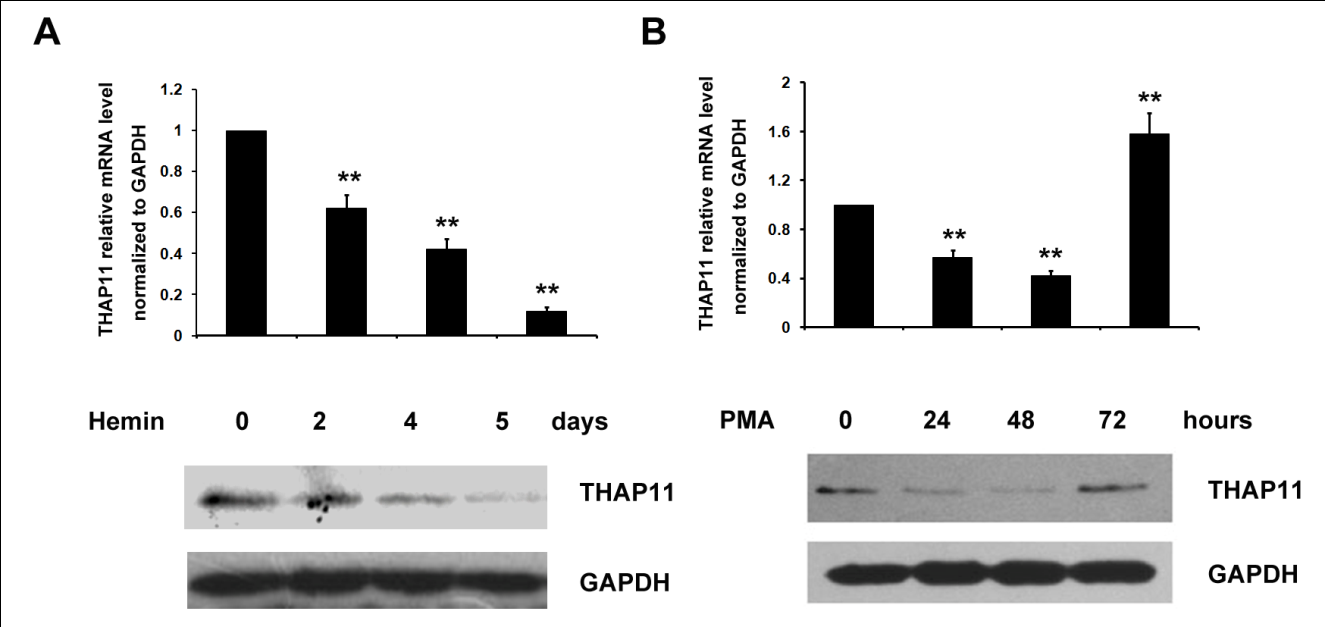


**Fig. S2 THAP11 expression profile during differentiation of K562 cells.** K562 cells were treated with (A) 40 μM hemin or (B) 10 nM PMA for the indicated time. Then the THAP11 expression level was analyzed using real-time PCR (upper panel) and Western blot analysis (lower panel). Real-time PCR results were expressed as fold induction relative to cells at day 0 and normalized to GAPDH mRNA. Each bar represented the mean ± SD for three independent experiments. The statistical difference between the samples was demonstrated as * P ≤ 0.05 or ** P ≤0.001. For Western blot analysis, GAPDH was used as internal control.
