# Supplementary material for: Effects of THAP11 on Erythroid Differentiation and Megakaryocytic Differentiation of K562 Cells
Source: PLoS One. 2014 Mar 17;9(3):e91557. doi: 10.1371/journal.pone.0091557 (PMC3956667; doi:10.1371/journal.pone.0091557)
Supplement: Figure S3 — THAP11 expression level in lentivirus-infected K562 cells. K562 cells were infected with control lentivirus (control) or THAP11 lentivirus (THAP11-LV) for twice in 48 hours. Then the GFP+ cells were sorted for Western blot analysis. GAPDH was used as internal control. exTHAP11: overexpressed THAP11; enTHAP11: endogenous THAP11. (DOCX) [file pone.0091557.s003.docx]

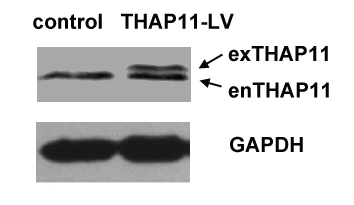


**Fig. S3 THAP11 expression level in lentivirus-infected K562 cells.** K562 cells were infected with control lentivirus (control) or THAP11 lentivirus (THAP11-LV) for twice in 48 hours. Then the GFP+ cells were sorted for Western blot analysis. GAPDH was used as internal control. exTHAP11: overexpressed THAP11; enTHAP11: endogenous THAP11.
