# Supplementary material for: Effects of THAP11 on Erythroid Differentiation and Megakaryocytic Differentiation of K562 Cells
Source: PLoS One. 2014 Mar 17;9(3):e91557. doi: 10.1371/journal.pone.0091557 (PMC3956667; doi:10.1371/journal.pone.0091557)
Supplement: Figure S4 — THAP11 expression levels in lentivirus-infected K562 cells during hemin-induced megakaryocytic differentiation. K562 cells were infected with control lentivirus (control) or THAP11 lentivirus (THAP11-LV) and GFP+ cells were purified. Then the cells were treated with 40 µM hemin for the indicated length of time and the THAP11 expression level was analyzed using Western blot analysis with anti-THAP11 antibody. GAPDH was used as internal control. exTHAP11: overexpressed THAP11; enTHAP11: endogenous THAP11. (DOCX) [file pone.0091557.s004.docx]

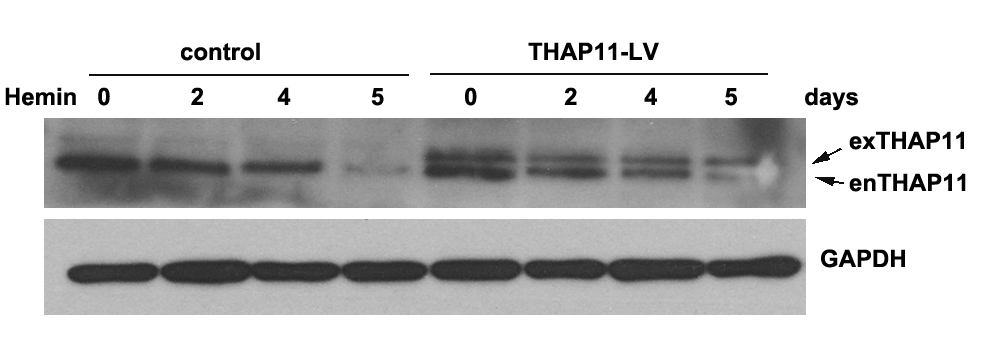


**Fig. S4. THAP11 expression levels in lentivirus-infected K562 cells during hemin-induced megakaryocytic differentiation.** K562 cells were infected with control lentivirus (control) or THAP11 lentivirus (THAP11-LV) and GFP+ cells were purified. Then the cells were treated with 40 μM hemin for the indicated length of time and the THAP11 expression level was analyzed using Western blot analysis with anti-THAP11 antibody. GAPDH was used as internal control. exTHAP11: overexpressed THAP11; enTHAP11: endogenous THAP11.
