# Supplementary material for: Effects of THAP11 on Erythroid Differentiation and Megakaryocytic Differentiation of K562 Cells
Source: PLoS One. 2014 Mar 17;9(3):e91557. doi: 10.1371/journal.pone.0091557 (PMC3956667; doi:10.1371/journal.pone.0091557)
Supplement: Figure S5 — THAP11 inhibits erythroid differentiation of human erythroleukemia cell line TF-1 induced by EPO. (A) TF-1 cells were infected with THAP11 lentivirus or control lentivirus and then cultured in the presence of 0.5 ng/ml GM-CSF and 5 IU/ml EPO for the indicated time. Then the benzidine positive cells were counted. The (B) HBA and (C) GPA mRNA levels were analyzed using real-time PCR. (D) THAP11 siRNA lentiviruses or control lentivirus were infected into TF-1 cells and cultured in the presence of 0.5 ng/ml GM-CSF and 5 IU/ml EPO for the indicated time. Then the benzidine-positive cells were counted. The (E) HBA and (F) GPA mRNA levels were analyzed using real-time PCR. (DOCX) [file pone.0091557.s005.docx]

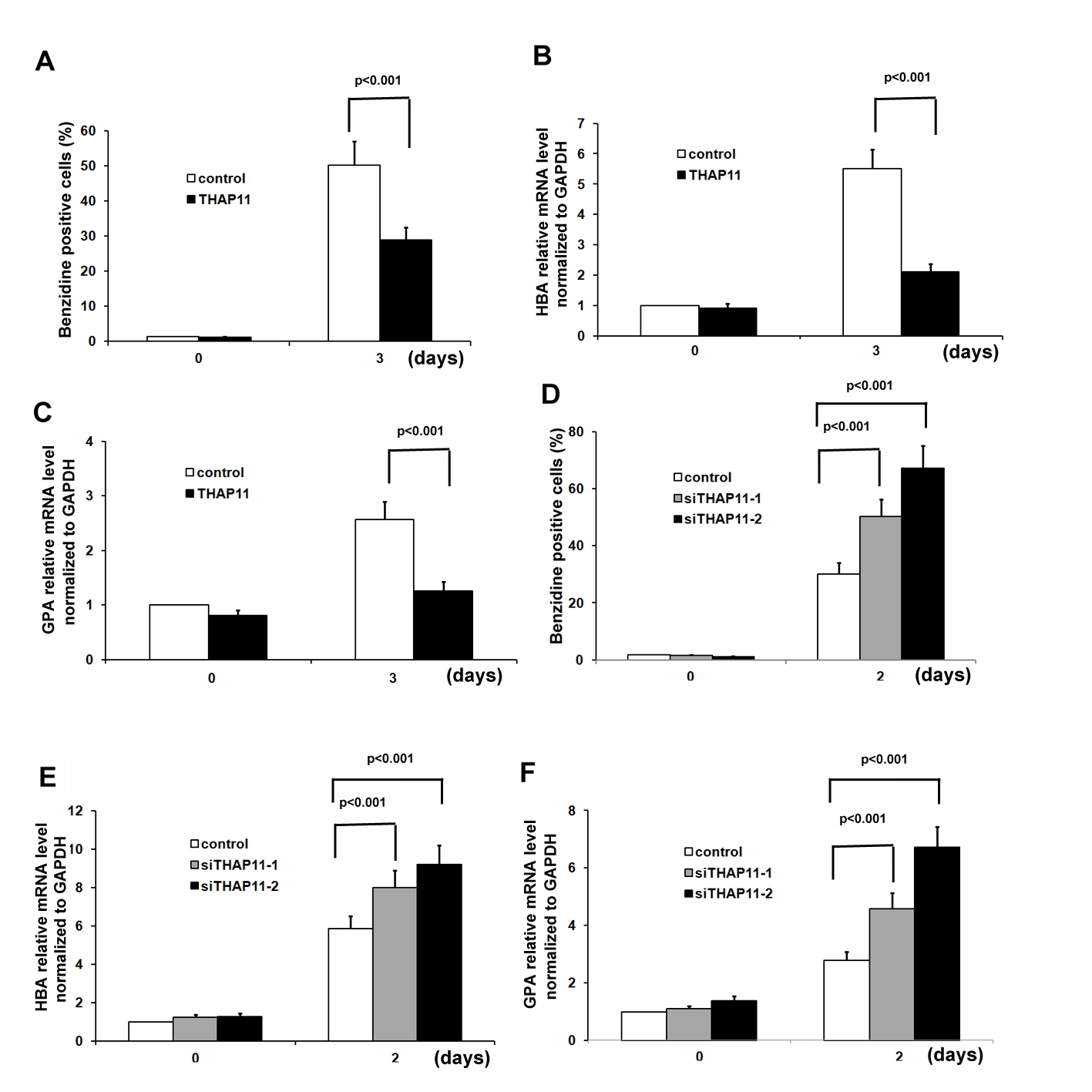


**Fig. S5. THAP11 inhibits erythroid differentiation of human erythroleukemia cell line TF-1 induced by EPO.** (A) TF-1 cells were infected with THAP11 lentivirus or control lentivirus and then cultured in the presence of 0.5 ng/ml GM-CSF and 5 IU/ml EPO for the indicated time. Then the benzidine positive cells were counted. The (B) HBA and (C) GPA mRNA levels were analyzed using real-time PCR. (D) THAP11 siRNA lentiviruses or control lentivirus were infected into TF-1 cells and cultured in the presence of 0.5 ng/ml GM-CSF and 5 IU/ml EPO for the indicated time. Then the benzidine-positive cells were counted. The (E) HBA and (F) GPA mRNA levels were analyzed using real-time PCR.
