# Supplementary material for: Effects of THAP11 on Erythroid Differentiation and Megakaryocytic Differentiation of K562 Cells
Source: PLoS One. 2014 Mar 17;9(3):e91557. doi: 10.1371/journal.pone.0091557 (PMC3956667; doi:10.1371/journal.pone.0091557)
Supplement: Figure S6 — The effect of THAP11 on H3 acetylation during PMA-indced megakaryocytic differentiation of K562 cells. K562 cells infected with THAP11-lentiviruses (THAP11-LV) and control cells were treated with 10 nM PMA for the indicated length of time. Then total cell lysates were prepared for analyzing the acetylation level of histone H3 using specific antibodies against acetylated-H3K9 and acetylated-H3K18. GAPDH was used as an internal control. (DOCX) [file pone.0091557.s006.docx]

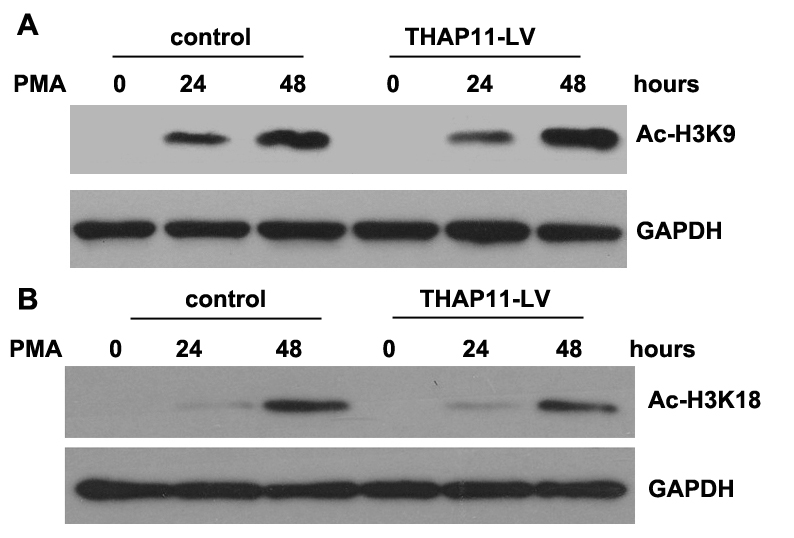


**Fig. S6. The effect of THAP11 on H3 acetylation during PMA-indced megakaryocytic differentiation of K562 cells.** K562 cells infected with THAP11-lentiviruses (THAP11-LV) and control cells were treated with 10 nM PMA for the indicated length of time. Then total cell lysates were prepared for analyzing the acetylation level of histone H3 using specific antibodies against acetylated-H3K9 and acetylated-H3K18. GAPDH was used as an internal control.
