# Supplementary material for: Effects of THAP11 on Erythroid Differentiation and Megakaryocytic Differentiation of K562 Cells
Source: PLoS One. 2014 Mar 17;9(3):e91557. doi: 10.1371/journal.pone.0091557 (PMC3956667; doi:10.1371/journal.pone.0091557)
Supplement: Figure S7 — Alteration of expression levels of several hematopoietic transcription factors in THAP11-overexpressing K562 cells with hemin treatment. K562 cells infected with THAP11-lentiviruses (THAP11-LV) and control cells were treated with 40 µM hemin for 72 hours. Then total RNA was extracted for real-time PCR analysis. Real-time PCR results were expressed as fold induction relative to cells at day 0 and normalized to GAPDH mRNA. Each bar represented the mean ± SD for three independent experiments. The statistical difference between the samples was demonstrated as * P≤0.05 or ** P≤0.001. (DOCX) [file pone.0091557.s007.docx]

**
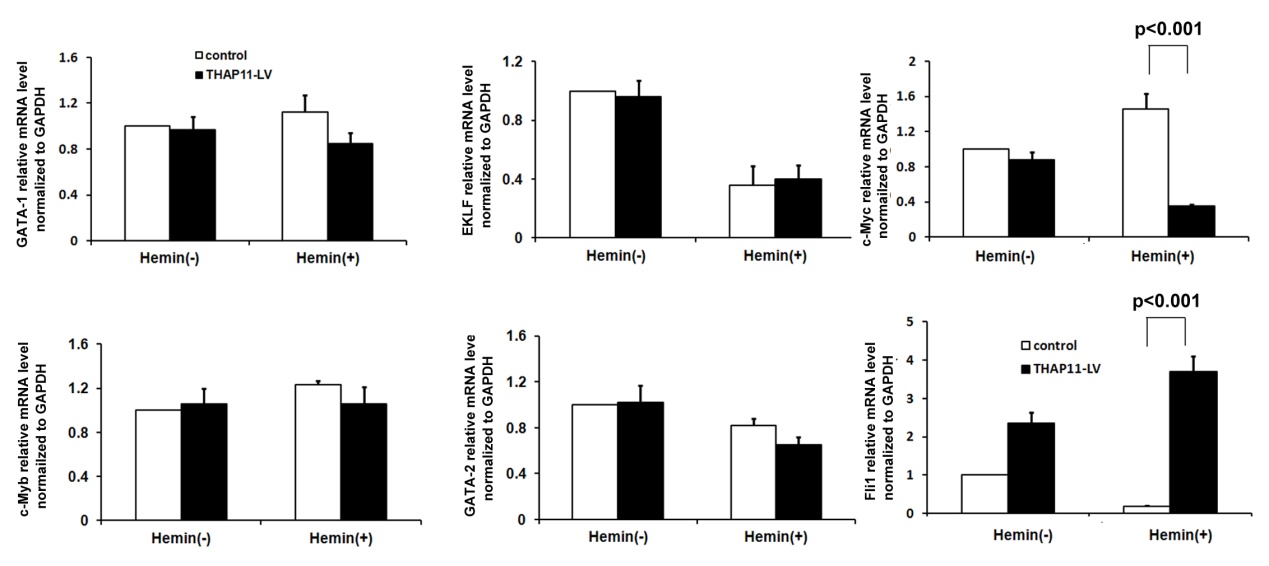
**

**Fig. S7 Alteration of expression levels of several hematopoietic transcription factors in THAP11-overexpressing K562 cells with hemin treatment.** K562 cells infected with THAP11-lentiviruses (THAP11-LV) and control cells were treated with 40 μM hemin for 72 hours. Then total RNA was extracted for real-time PCR analysis. Real-time PCR results were expressed as fold induction relative to cells at day 0 and normalized to GAPDH mRNA. Each bar represented the mean ± SD for three independent experiments. The statistical difference between the samples was demonstrated as * P ≤ 0.05 or ** P ≤0.001.
