# Supplementary material for: Effects of THAP11 on Erythroid Differentiation and Megakaryocytic Differentiation of K562 Cells
Source: PLoS One. 2014 Mar 17;9(3):e91557. doi: 10.1371/journal.pone.0091557 (PMC3956667; doi:10.1371/journal.pone.0091557)
Supplement: Figure S8 — Bioinformatic analysis of the promoter sequences of GATA2 (A), c-Myb (B) and Fli1 (C) genes. The potential THAP11 binding consensus sequences are framed. (DOCX) [file pone.0091557.s008.docx]

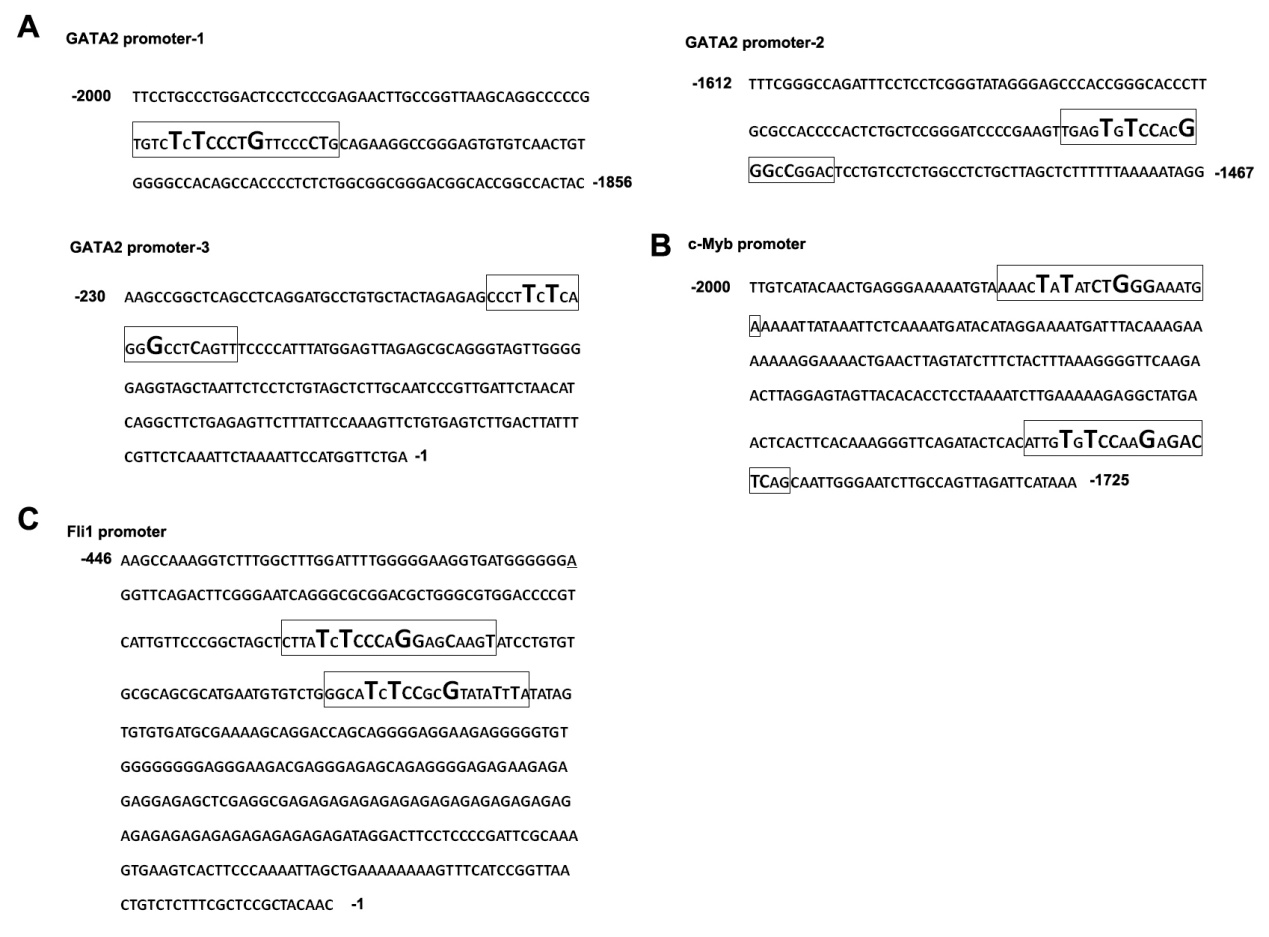


**Fig. S8 Bioinformatic analysis of the promoter sequences of GATA2 (A) , c-Myb (B) and Fli1 (C) genes.** The potential THAP11 binding consensus sequences are framed.
