# Supplementary material for: Effects of THAP11 on Erythroid Differentiation and Megakaryocytic Differentiation of K562 Cells
Source: PLoS One. 2014 Mar 17;9(3):e91557. doi: 10.1371/journal.pone.0091557 (PMC3956667; doi:10.1371/journal.pone.0091557)
Supplement: Figure S9 — THAP11 occupies the promoter regions of GATA2, c-Myb and Fli1 genes. (A) ChIP at the promoter region of indicated genes using anti-THAP11 antibody and IgG as the control in K562 cells. A random probe was used from human GAPDH genome. (B) The results are averages of at least 3 independent experiments. Error bars represent the mean ± SD. Statistical significance was determined by comparing the occupancy of specific antibodies and the IgG control; *P≤0.05, ** p<0.001. (DOCX) [file pone.0091557.s009.docx]

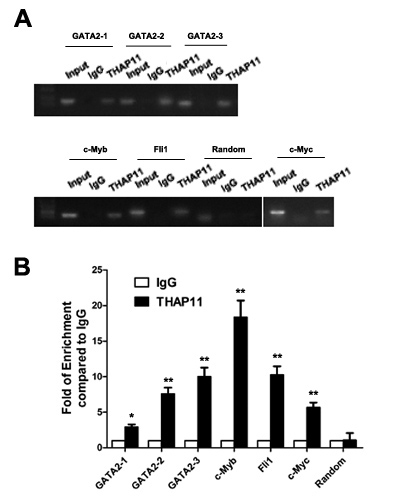


**Fig. S9 THAP11 occupies the promoter regions of GATA2, c-Myb and Fli1 genes.** (A) ChIP at the promoter region of indicated genes using anti-THAP11 antibody and IgG as the control in K562 cells. A random probe was used from human GAPDH genome. (B) The results are averages of at least 3 independent experiments. Error bars represent the mean ± SD. Statistical significance was determined by comparing the occupancy of specific antibodies and the IgG control; *P≤0.05, ** p<0.001.
