# Supplementary material for: Effects of THAP11 on Erythroid Differentiation and Megakaryocytic Differentiation of K562 Cells
Source: PLoS One. 2014 Mar 17;9(3):e91557. doi: 10.1371/journal.pone.0091557 (PMC3956667; doi:10.1371/journal.pone.0091557)
Supplement: Table S1 — Sequences of primers used in the present study. (DOC) [file pone.0091557.s010.doc]

**Table S1. Sequences of primers used in the present study**

| primer | Sequence (5’-3’) | target | Use |
| --- | --- | --- | --- |
| SiTHAP11-1 | TGC GCT GCA CTT CTA CAC GTT TCA AGA GAA CGT GTA GAA GTG CAG CGC TTT TTT C | THAP11 | siRNA lentivirus |
| SiTHAP11-2 | TGA CCA TTC GTA CTC CTT GTT TCA AGA GAA CAA GGA GTA CGA ATG GTC TTT TTT C | THAP11 | siRNA lentivirus |
| GPA-F  GPA-R | GACAAATGATACGCACAAACGG  TCCAATAACACCAGCCATCAC | GPA  GPA | Real-time PCR  Real-time PCR |
| HBA-F | CAACTTCAAGCTCCTAAGCC | HBA | Real-time PCR |
| HBA-R | CTTAACGGTATTTGGAGGTCAG | HBA | Real-time PCR |
| EKLF-F | ATGACTTCCTCAAGTGGTGG | EKLF | Real-time PCR |
| EKLF-R | CTCTCATCGTCCTCTTCCTC | EKLF | Real-time PCR |
| THAP11-F | AGAAGACGTGAAGCCCATCGA | THAP11 | Real-time PCR |
| THAP11-R | TGGTGCCTGACGACAAGGAGTA | THAP11 | Real-time PCR |
| c-Myb-F | TTCTGAAGCACAAAATGTCTCCA | c-Myb | Real-time PCR |
| c-Myb-R | CCCACATAATGGTAGCACCTG | c-Myb | Real-time PCR |
| GATA2-F | ATCCACCCTTCCTCCAGTCT | GATA-2 | Real-time PCR |
| GATA2-R | CGGGAGCCAAGAGTATGTTC | GATA-2 | Real-time PCR |
| GATA1-F | GCACCAACTGCCAGACGACC CAGATGCCTTGCGGTTTCGA | GATA-1  GATA-1 | Real-time PCR  Real-time PCR |
| GATA1-R |
| Fli1-F | CCCACCAGCAGAAGGTGAAC  ATGCGGCTCCAAAGAAGCT | Fli1  Fli1 | Real-time PCR  Real-time PCR |
| Fli1-R |
| CD61-F | TGTATGGGACTCAAGATTGGA | CD61 | Real-time PCR |
| CD61-R | AGCGATGGCTATTAGGTTCA | CD61 | Real-time PCR |
| c-Myc -F | CGTCTCCACACATCAGAGCACAA | c-Myc | Real-time PCR |
| c-Myc -R | GCAGCAGGATAGTCCTT | c-Myc | Real-time PCR |
| GAPDH-F | AACGTGTCAGTGGTGGACCT | GAPDH | Real-time PCR |
| GAPDH-R | TGCTGTAGCCAAATTCGTTG | GAPDH | Real-time PCR |
| GATA-2-1F | CTGGACTCCCTCCCGAGAACTT | GATA-2 | ChIP-qPCR |
| GATA-2-1R | GAGTGGTCGGTTCTGCCCATTC | GATA-2 | ChIP-qPCR |
| GATA-2-2F | GCCAGATTTCCTCCTCGGGTAT | GATA-2 | ChIP-qPCR |
| GATA-2-2R | GGGCTGAGCCACAAGGAAAAGT | GATA-2 | ChIP-qPCR |
| GATA-2-3F | AGCCTCAGGATGCCTGTGCTACTA | GATA-2 | ChIP-qPCR |
| GATA-2-3R | GGGATTGCAAGAGCTACAGAGGAG | GATA-2 | ChIP-qPCR |
| c-Myb F | TTGTCATACAACTGAGGGAAAAATG | c-Myb | ChIP-qPCR |
| c-Myb R | TTATGAATCTAACTGGCAAGATTCC | c-Myb | ChIP-qPCR |
| Fli1 F | AGGTTCAGACTTCGGGAATCAGGG | Fli1 | ChIP-qPCR |
| Fli1 R | GCTGGTCCTGCTTTTCGCATCACA | Fli1 | ChIP-qPCR |
| c-Myc F | TAATCATTCTAGGCATCGTTTT | c-Myc | ChIP-qPCR |
| c-Myc R | ATCATCGCAGGCGGAACAGCTG | c-Myc | ChIP-qPCR |
| GAPDH F | CTCCTGCACCACCAACTGCTTAGC | GAPDH | ChIP-qPCR |
| GAPDH R | CCATCACGCCACAGTTTCC | GAPDH | ChIP-qPCR |
